# Posttreatment FDG PET/CT in predicting survival of patients with ovarian carcinoma

**DOI:** 10.1186/s13550-016-0194-7

**Published:** 2016-05-20

**Authors:** Linda C. Chu, Hua-Ling Tsai, Hao Wang, John Crandall, Mehrbod S. Javadi, Richard L. Wahl

**Affiliations:** Russell H. Morgan Department of Radiology and Radiological Science, Johns Hopkins University School of Medicine, 1800 Orleans Street, Baltimore, MD 21287 USA; Johns Hopkins Division of Biostatistics and Bioinformatics, Johns Hopkins University School of Medicine, 550 North Broadway, Baltimore, MD 21287 USA; Mallinckrodt Institute of Radiology, Washington University School of Medicine, Campus Box 8131, 660S. Euclid Avenue, St. Louis, MO 63110 USA

**Keywords:** Ovarian carcinoma, PET/CT, CA-125, Patient survival, Prognosis

## Abstract

**Background:**

The purposes of this study are to evaluate the prognostic value of posttreatment ^18^F-FDG PET/CT in predicting the survival of patients with ovarian carcinoma and to determine incremental value of combining posttreatment PET/CT with traditional prognostic factors in a multivariate model.

**Methods:**

This was an IRB-approved retrospective study. From July 2001 to July 2011, 48 patients who completed initial therapy for ovarian carcinoma with concurrent 3- to 9-month initial posttreatment ^18^F-FDG PET/CT and serum CA-125 were identified from the radiology database. Prognostic value of posttreatment PET/CT, CA-125, age, race, and tumor stage were determined from Cox proportional hazard model using univariate and multivariate analyses. Time-dependent receiver operator curves were also calculated at various follow-up intervals.

**Results:**

In a univariate model, overall survival (OS) was associated with PET/CT (hazard ratio = 4.18; 95 % CI 1.49–11.70) and CA-125 (hazard ratio = 11.09; 95 % CI 4.27–28.79). When the effects of posttreatment PET/CT and CA-125 were combined in the multivariate analysis, hazard ratio for PET/CT increased to 4.84 (95 % CI 1.59–14.73, *p* = 0.005) and hazard ratio for CA-125 increased to 14.43 (95 % CI 4.65–44.84, *p* < 0.001). In the subset of patients with negative CA-125, posttreatment PET/CT had a hazard ratio of 2.98 (95 % CI 0.86–10.37), supporting the role of posttreatment PET/CT in risk stratification of patients with negative CA-125. Time-dependent receiver operator curves showed that the combination of PET/CT and CA-125 improved prognostic accuracy compared to PET/CT or CA-125 alone at 12-, 24-, 30-, and 36-month follow-up.

**Conclusions:**

Posttreatment PET/CT can predict the survival of patients with ovarian carcinoma. The addition of posttreatment PET/CT to the CA-125 serum biomarker has an incremental value in improving prognostic accuracy, particularly in the subset of patients with negative CA-125.

## Background

Ovarian cancer is diagnosed in 65,500 women each year in Europe and results in 42,700 deaths yearly [1]. Although responsible for <30 % of all gynecologic malignancies, ovarian cancer accounts for >50 % of deaths [2]. Most patients are diagnosed at advanced stage [3], and 5-year survival for ovarian cancer has been reported to be between 35 and 44 % [3, 4]. Primary cytoreductive surgery followed by taxane and platinum-based combination chemotherapy is the mainstay of treatment for women with advanced stage ovarian cancer [5, 6]. Despite achievement of complete clinical response, recurrence rates remain high. ^18^F-fluorodeoxyglucose positron emission tomography/computed tomography (^18^F-FDG PET/CT) has been shown to detect active disease at relatively low levels of CA-125, thereby facilitating early diagnosis of recurrence or residual disease [7]. The reported sensitivity and specificity of PET alone and PET/CT in the detection of recurrent ovarian cancer range from 45.3 to 92.3 % and 73.0 to 100 % [8–12]. PET/CT has been shown to alter clinical management in patients with suspected recurrent ovarian cancer in 60 % of cases [13]. Pretreatment PET/CT has been shown to predict disease recurrence and overall survival in patients with ovarian cancer [14–16]. Evangelista et al. and Liao et al. have shown that posttreatment PET/CT obtained between 1 and 109 months after the treatment for ovarian cancer was helpful in predicting patient survival [17, 18]. However, Kurosaki et al. showed that posttreatment PET/CT was not significant in predicting patient survival [19]. Currently, the prognostic impact of surveillance posttreatment PET/CT and the ideal time window for surveillance imaging have not been firmly established. In fact, current guidelines by the National Comprehensive Cancer Network (NCCN) and the Society of Gynecologic Oncologists state that there is insufficient evidence to support the use of PET/CT in routine surveillance [2, 6]. The purposes of this study are to evaluate the prognostic impact of surveillance posttreatment PET/CT in predicting survival of ovarian cancer patients and to compare the prognostic value of PET/CT with CA-125 in predicting patient survival. The potential prognostic impact of posttreatment PET/CT may support its more widespread use in routine surveillance.

## Methods

### Patient population

This is an Institutional Review Board-approved retrospective study. From July 2001 to July 2011, 48 patients (mean age of diagnosis 58.1 years) who completed initial therapy for ovarian carcinoma with no known cancer recurrence who underwent concurrent PET/CT and serum CA-125 within 3 to 9 months of completion of primary therapy for ovarian carcinoma were identified from the radiology database (Table 1). Complete response to initial therapy was assessed based on normalization of CA-125 levels and the absence of suspicious findings on posttreatment CT within 1–2 months after completion of treatment. At our institution, patients usually returned for clinical follow-up after initial treatment every 2 to 4 months for the first 2 years. Complete history, physical exam, and CA-125 were obtained at each clinical visit. CT and PET/CT were obtained as clinically appropriate for restaging in asymptomatic patients and to evaluate for suspected recurrence based on clinical findings and/or abnormal CA-125. The time window of 3 to 9 months was chosen as many ovarian carcinoma patients presented for follow-up PET/CT around 6 months posttreatment. 3 to 9 months gave a 3-month buffer window centered on the 6-month follow-up scan. Three of the 48 patients underwent more than one PET/CT exam during the 3- to 9-month time window, and the latest PET/CT was used for the analysis.

**Table 1:** Patient demographics and clinical characteristics (*n* = 48)

Characteristic	*N* (range or %)
Median (range) of age at diagnosis (years)	58.0 (35–85)
Median (range) between completion of initial treatment and PET/CT (months)	6 (3–9)
Mean ± SD between completion of initial treatment and PET/CT (months)	5.5 ± 1.9
Median follow-up time^a^ (months)	47.3 (4.56–119.8)
Death events	22 (45.8 %)
Median baseline CA-125 (U/mL)	459 (14–8409)
Median CA-125 at follow-up (U/mL)	23.5 (2–626)
FIGO stage	
I and II	4 (8.33 %)
III and IV	43 (89.58 %)
Unknown	1 (2.08 %)
Histologic type	
Serous	35 (72.92 %)
Not serous	12 (25.00 %)
Unknown	1 (2.08 %)
Tumor grade	
High	40 (83.33 %)
Low	2 (4.17 %)
Unknown	6 (12.50 %)
Residual disease	
Optimal debulking	32 (66.67 %)
Suboptimal debulking	3 (6.25 %)
Unknown	13 (27.08 %)
Initial treatment	
Surgery	48 (100 %)
Adjuvant chemotherapy	42 (87.5 %)
Radiation therapy	0 (0 %)

^a^Median follow-up was calculated via reverse Kaplan-Meier method

### PET/CT scan technique

Patients fasted for a minimum of 4 h and had blood glucose levels less than or equal to 200 mg/dL just before the intravenous injection of ^18^F-FDG (8.14 MBq/kg [0.22 mCi/kg]). Oral contrast was administered for the CT portion of the study. After an uptake phase of ^18^F-FDG of approximately 60 min, a combined whole body PET/CT scan (Discovery LS; GE Healthcare) was performed. Single whole body CT was performed with a four-slice multidetector helical scanner with oral contrast (no IV contrast) for attenuation correction purpose. A CT transmission map was generated for image fusion. Emission data were acquired for 5 min at each bed position. PET images were reconstructed using the ordered-subset expectation maximization algorithm (2 iterations, 28 subsets), an 8-mm Gaussian filter with a 128 × 128 matrix, and non-contrast-enhanced CT attenuation correction.

### PET/CT and CA-125 data

PET/CT reports were systematically reviewed to determine the presence or absence of suspected disease recurrence on PET/CT. PET/CT exams were interpreted by junior and senior board-certified nuclear medicine physicians ranging from 2 years to greater than 10 years of experience. Reports that stated “findings compatible with disease recurrence” or “findings suspicious for disease recurrence” were classified as presence of suspected disease recurrence. Reports that stated “no evidence of disease recurrence” or “findings most likely representing posttreatment changes, with disease recurrence not entirely excluded” were classified as absence of suspected disease recurrence.

Medical records were reviewed to determine concurrent serum CA-125 within 1 month of PET/CT. Recurrence based on serum CA-125 was determined based on the Gynecological Cancer InterGroup (GCIG) criteria: In patients with elevated CA-125 pretreatment and normalization of CA-125, recurrence was determined by CA-125 greater than, or equal to, two times the upper limit of the reference range on two occasions at least 1 week apart. In patients with elevated CA-125 before treatment, which never normalized, recurrence was determined by CA-125 greater than, or equal to, two times the nadir value on two occasions at least 1 week apart [20].

The sensitivity and specificity in detection of disease recurrence with initial posttreatment PET/CT and CA-125 were compared to reference standard 6 months later. Reference standard included pathologic confirmation, elevated CA-125, and/or stable of increase in size of FDG avid lesion on follow-up PET/CT.

### Survival data

Patient mortality data was determined from the medical record and social security death index. Overall survival was defined from the completion of initial treatment to death or censored at last follow-up date, up until 31 December 2014.

### Statistical analysis

Kaplan-Meier curve was plotted by PET/CT results, by CA-125 results, and by combination of PET/CT and CA-125 results. Cox proportional hazard model was used to evaluate the association of the prognostic factors with survival. Univariate analysis of overall survival was performed to examine the effects of PET scan, CA-125, age, race, and initial tumor stage. These factors were then carried on to a multivariate analysis. The ability of the prognostic factor to distinguish between patients differing in survival was assessed using the C-statistic. The C-statistic represents the probability that for a randomly selected pair of patients, the one having higher predicted survival is the one who survives longer. In addition, area under the curve (AUC) from time-dependent receiver operator curve (ROC) via nearest neighbor estimation [21] for predicting survival at the time of interests (1, 2, 2.5, and 3 years) was considered as well. The larger value of C-statistic and AUC indicates a better discrimination ability of the prognostic performance. Statistical software R 3.2.0 was performed through the analyses, and statistical packages “Hmisc” and “survivalROC” were applied for evaluating C-statistic and time-dependent ROC.

## Results

**Fig. 1:**
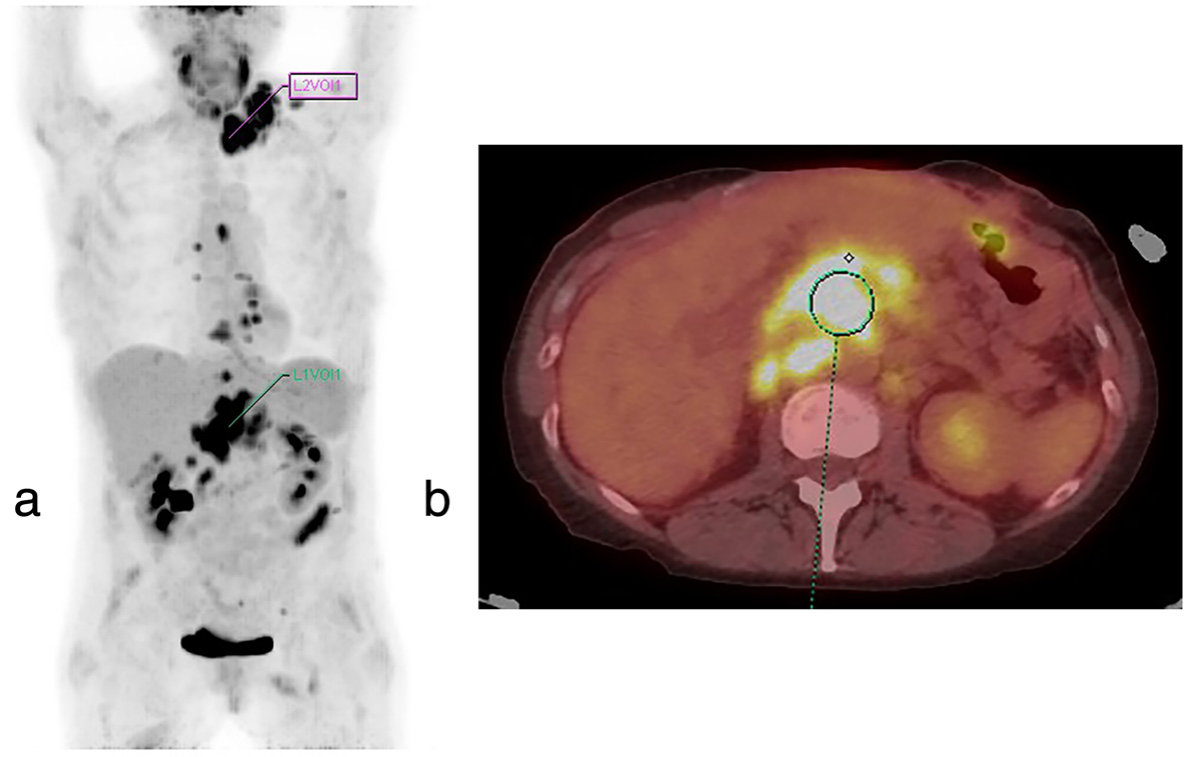
PET/CT of a 52-year-old ovarian cancer patient status post debulking and chemotherapy with active residual disease. Concurrent CA-125 was 89. **a** PET maximum intensity projection (MIP) image shows intensely FDG avid disease in the left supraclavicular lymph nodes, mediastinal lymph nodes, retroperitoneal lymph nodes, and peritoneal implants. **b** Axial fused PET/CT image shows cluster of FDG avid retroperitoneal lymph nodes. Volume of interest shows SUV_max_ of 8.67

**Fig. 2:**
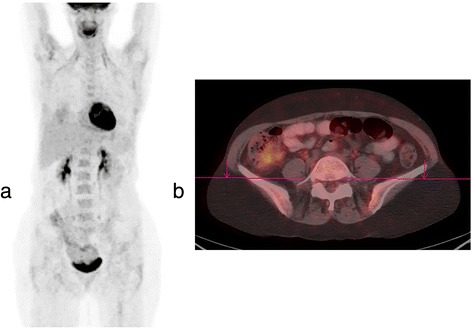
PET/CT of a 54-year-old ovarian cancer patient status post debulking and chemotherapy with no evidence of active residual disease. Concurrent CA-125 was 5. **a** PET MIP image shows physiologic FDG uptake without evidence of active residual disease. **b** Axial-fused PET/CT image shows physiologic intestinal FDG uptake in the *right lower quadrant* and the pelvis.

Patient demographics and clinical characteristics are summarized in Table 1. Median time between the completion of initial treatment and PET/CT was 6 months (range 3 to 9 months; mean 5.5 ± 1.9 months). Twenty four patients were asymptomatic with no clinical signs of recurrence at time of exam. Twenty four patients had suspected recurrence based on clinical exam, CT findings, or elevated CA-125. Posttreatment PET/CT reports showed findings “compatible with” or “suspicious for” recurrence in 62.5 % (30/48) cases. Representative positive and negative PET/CT exams were shown in Figs. 1 and 2. Concurrent CA-125 was suspicious for recurrence in 27.1 % (13/48) cases.

Both PET/CT and CA-125 were suspicious for recurrence in 25.0 % (12/48) cases. Both PET/CT and CA-125 were negative for recurrence in 35.4 % (17/58) cases. PET/CT and CA-125 were discordant for recurrence in 39.6 % (19/48) cases, in which PET/CT was positive and CA-125 was negative in 37.5 % (18/48) cases and PET/CT was negative and CA-125 was positive in 2.1 % (1/48) cases. In the 18 cases where PET/CT was positive and CA-125 was negative, baseline CA-125 was elevated in 61.1 % (11/18) cases, baseline CA-125 was within normal limits in 11.1 % (2/18) cases, and baseline CA-125 was unknown in 27.8 % (5/18) cases.

Forty one out of 48 (85.4 %) patients received additional surgical resection and/or adjuvant chemotherapy treatment following initial posttreatment PET/CT. The sensitivity and specificity in detection of disease recurrence with initial posttreatment PET/CT and CA-125 were compared to reference standard 6 months later. Reference standard showed evidence of disease recurrence in 79.2 % (38/48) of patients 6 months after the initial posttreatment follow-up. It was based on pathologic confirmation in 45.8 % (22/48) cases. It was based on clinical assessment in 54.2 % (26/48) cases, which was defined as positive in the setting of persistently elevated CA-125, and/or stable or increase in lesion size of follow-up CT or PET/CT. PET/CT showed sensitivity of 78.9 % (30/38, 95 % CI 62.7–90.4 %), specificity of 100 % (10/10, 95 % CI 58.7–100.0 %), and accuracy of 83.3 % (40/48, 95 % CI 69.8–92.5 %). CA-125 showed sensitivity of 34.2 % (13/38, 95 % CI 19.6–51.4 %), specificity of 100 % (10/10, 95 % CI 58.7–100.0 %), and accuracy of 47.9 % (23/48, 95 % CI 33.3–62.8 %).

**Fig. 3:**
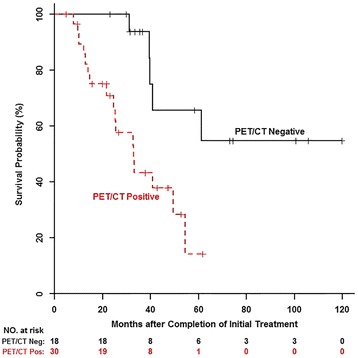
Kaplan-Meier overall survival curve by ^18^F-FDG PET/CT

**Fig. 4:**
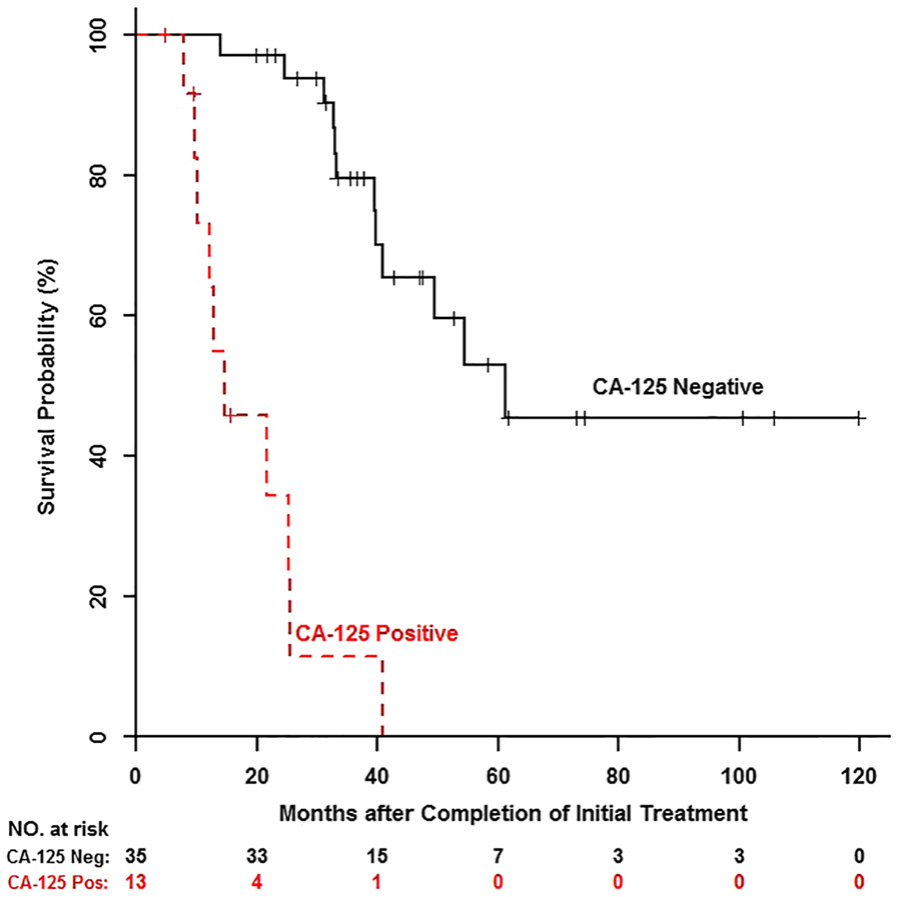
Kaplan-Meier overall survival curve by CA-125

In the univariate analysis, PET/CT and serum CA-125 were statistically significant in the prediction of overall survival (Table 2). The hazard ratio and C-statistic for PET/CT were 4.18 (95 %CI 1.49–11.70) and 0.683, respectively (*p* = 0.006). The hazard ratio and C-statistic for CA-125 were 11.09 (95 % CI 4.27–28.79) and 0.744, respectively (*p* < 0.001). The Kaplan-Meier overall survival curves showed improved patient survival in patients with negative posttreatment PET/CT (Fig. 3) or negative posttreatment CA-125 (Fig. 4).

**Table 2:** Univariate overall survival analysis with age at diagnosis, race, and initial tumor stage, PET/CT, and serum CA-125 and multivariate overall survival analysis with PET/CT and serum CA-125

	Hazard ratio	Lower CI^a^	Upper CI^a^	*p* value	C-statistic
Univariate analysis					
Age	1.03	0.98	1.07	0.277	0.509
Race (White vs. non-White)	0.53	0.16	1.82	0.316	0.518
Stage (stage ≥III vs. stage <III)	1.02	0.24	4.41	0.975	0.513
Baseline CA-125 (log transformation)	0.90	0.48	1.68	0.73	0.58
PET/CT (+ vs. −)	4.18	1.49	11.70	0.006	0.683
CA-125 (+ vs. −)	11.09	4.27	28.79	<0.001	0.744
Multivariate analysis					
PET/CT (+ vs. −)	4.84	1.59	14.73	0.005	0.804
CA-125 (+ vs. −)	14.43	4.65	44.84	<0.001	

^a^Denotes confidence interval

Multivariate analysis was performed including both PET/CT and CA-125 to determine if the addition of PET/CT to CA-125 would improve prognostic accuracy. In the multivariate analysis model including both PET/CT and CA-125, the hazard ratio for PET/CT increased to 4.84 (95 % CI 1.59–14.73) (*p* = 0.005) and the hazard ratio for CA-125 increased to 14.43 (95 % CI 4.65–44.84) (*p* < 0.001). C-statistic increased to 0.804 (Table 2).

**Fig. 5:**
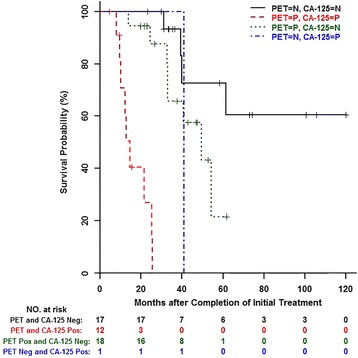
Kaplan-Meier overall survival curve by combination of ^18^F-FDG PET/CT and concurrent CA-125; *N* negative, *P* positive

For exploratory manner, in the subset of 35 patients with negative CA-125, univariate analysis with posttreatment PET/CT had a hazard ratio of 2.98 (95 % CI 0.86–10.37) and C-statistic of 0.641, with *p* value of 0.086, supporting the role of posttreatment PET/CT in risk stratification of patients with negative CA-125. Kaplan-Meier overall survival curve combining both posttreatment PET/CT and CA-125 (Fig. 5) showed that patients with positive PET/CT and positive CA-125 had the lowest likelihood of overall survival and patients with negative PET/CT and negative CA-125 had the highest likelihood of overall survival. Patients with either positive PET/CT and negative CA-125 or negative PET/CT and positive CA-125 had intermediate likelihood of overall survival. Posttreatment PET/CT may play a role in risk stratification in patients with negative CA-125, although a larger cohort will be needed for further validation.

**Fig. 6:**
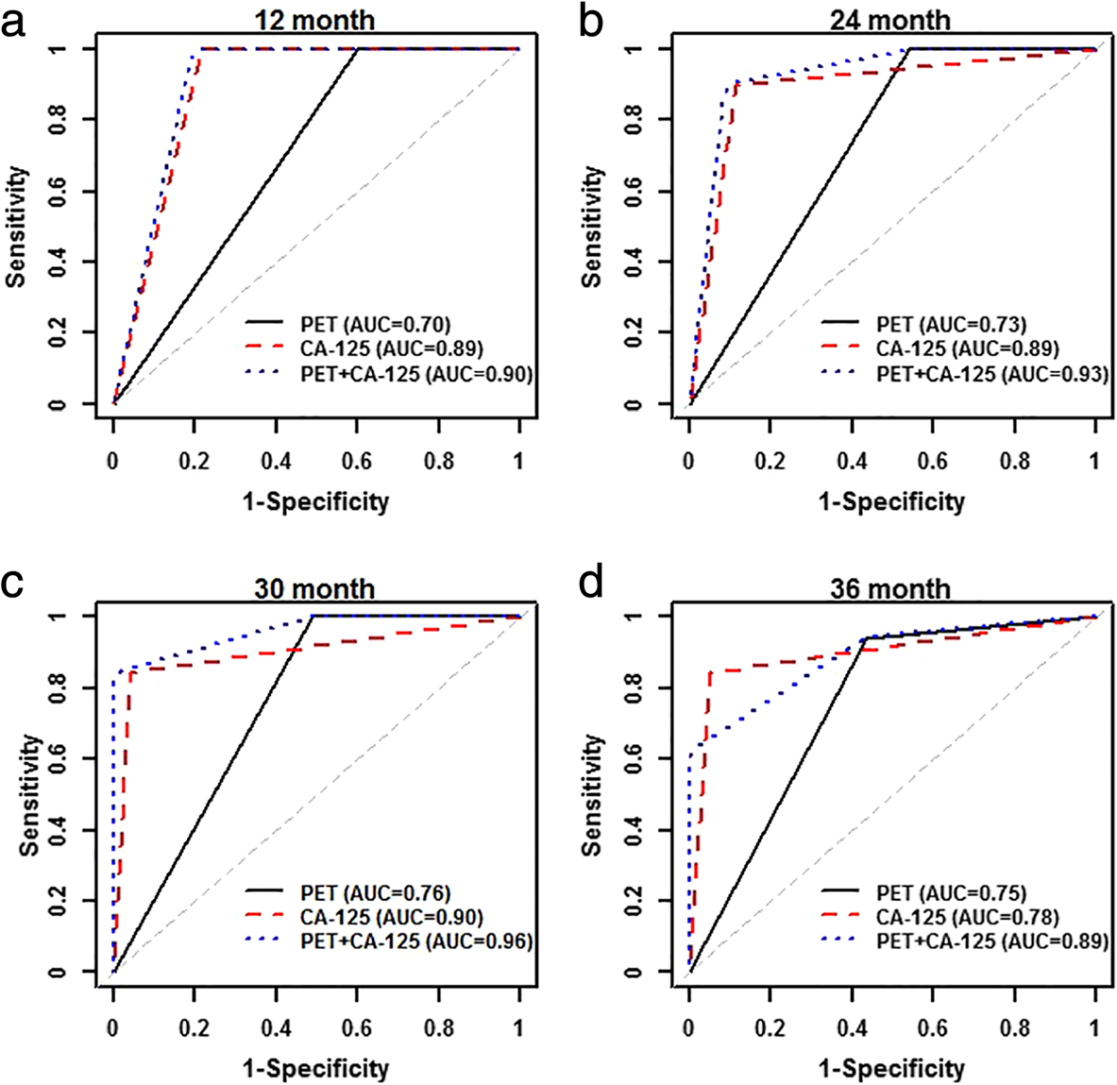
Time-dependent receiver operator curves on predicting survival with PET/CT, CA-125, and combination of PET/CT and CA-125, at **a** 12, **b** 24, **c** 30, and **d** 36 months

The time-dependent receiver operator curve analysis also showed that adding PET/CT to CA-125 increased the area under the ROC compared to PET/CT alone or CA-125 alone at each of the 12-, 24-, 30-, and 36-month follow-up time points (Fig. 6). For example, at the 12-month follow-up, area under the receiver operator curve was 0.90 for combination of PET/CT and CA-125, compared with 0.70 for PET/CT alone and 0.89 for CA-125 alone. Receiver operator curves at 24-, 30-, and 36-month follow-up showed similar results, with more increments compared to CA-125 alone.

## Discussion

Posttreatment PET/CT has been shown to be sensitive and specific in the detection of recurrent ovarian cancer [8–12], and it has significant impact in altering clinical management in patients with suspected recurrent ovarian cancer [13]. Our results showed that initial posttreatment PET/CT within 3 to 9 months was more sensitive than CA-125 in detection of disease recurrence. PET/CT detected disease recurrence in 62.5 % of ovarian cancer patients within 9 months of completing initial therapy. This percentage may be higher than expected for several reasons. PET/CTs were not routinely obtained in all ovarian cancer patients within the first 9 months of posttreatment at our institution. Our clinicians selected a subset of patients to undergo surveillance PET/CT based on clinical findings or CA-125 levels that place patients at increased risk of disease recurrence. As a result, 50 % of our patients have suspected ovarian cancer recurrence at the time of restaging PET/CT. In addition, 6.25 % of patients were known to have suboptimal debulking at the time of surgery and 27.1 % of patients had surgeries performed at outside institutions and it was unknown in how many cases optimal debulking was achieved.

Similar to previously published reports, patients with either positive posttreatment PET/CT or positive CA-125 had worse prognosis compared with patients with negative posttreatment PET/CT and negative CA-125 [17, 18, 22]. Initial posttreatment CA-125 was more predictive of overall survival than PET/CT, and this trend was consistent across all of our statistical analyses, including Cox proportional hazard model, C-statistic, Kaplan-Meier, and time-dependent receiver operator curves. The addition of posttreatment PET/CT to serum CA-125 may be valuable in risk stratification of the subset of patients with negative CA-125, which may be further validated in a larger cohort. Previous reports investigating effects of posttreatment PET/CT and patient prognosis used studies ranging from 1 to 109 months posttreatment [17, 19]. At our institution, patients usually would have undergone multiple posttreatment PET/CTs during that time period, and they may have both positive and negative PET/CTs rendering the results difficult to interpret. We chose to focus our population on the initial posttreatment PET/CT within 3 to 9 months as patients often returned for follow-up PET/CT at this time interval. Focusing on this specific time period allows us to determine the prognostic value of the first initial PET/CT most relevant to our patient population.

Due to the retrospective nature of this study, patients with positive PET/CT and/or CA-125 received different treatments, including additional surgical debulking, chemotherapy, or conservative watchful waiting. There has been conflicting data whether treatment of early recurrence in asymptomatic patients (based on CA-125 and/or imaging) with traditional surgical debulking or chemotherapy would lead to increased survival [23]. A small retrospective study showed improved overall survival in asymptomatic patients whose disease was detected by routine surveillance testing [24]. However, two larger studies showed no survival benefit with early treatment of relapsed based on raised CA-125 level alone [25] or based on CA-125 levels and/or imaging [26]. More recently, several clinical trials have shown that bevacizumab, an angiotensin inhibitor, was able to prolong progression-free survival in patients with advanced stage ovarian carcinoma [27–30]. Bevacizumab has also been shown to increase overall survival in the subset of patients with high risk of disease progression [31]. With its high sensitivity in detection of disease recurrence, posttreatment PET/CT may be able to identify this subset of patients with high risk of disease progression who would benefit most from these newer treatments.

In the current pilot study, a time window of 3 to 9 months was chosen as ovarian carcinoma patients at our institution often returned for follow-up PET/CT at this time interval. In future studies, the prognostic value of posttreatment PET/CT and serum CA-125 can be determined at different time intervals to determine the ideal time window for posttreatment surveillance. In future studies, it may be helpful to determine if the quantitative analysis can refine prognostic value of PET/CT.

## Conclusions

Posttreatment PET/CT can predict survival of patients with ovarian carcinoma. Addition of posttreatment PET/CT to the CA-125 serum biomarker has an incremental value in improving prognostic accuracy, particularly in the subset of patients with negative CA-125.

### Ethics approval and consent to participate

All procedures performed in studies involving human participants were in accordance with the ethical standards of the institutional and/or national research committee and with the 1964 Helsinki Declaration and its later amendments or comparable ethical standards. For this type of study, formal consent is not required.

### Availability of data and materials

The dataset supporting the conclusions of this article with the supplementary materials.

## Abbreviations

PET/CT: positron emission tomography/computed tomography
NCCN: National Comprehensive Cancer Network
GCIG: Gynecological Cancer InterGroup
AUC: area under the curve
ROC: receiver operator curve
SUV: standardized uptake value
MIP: maximum intensity projection
